# New Device Measures Atmospheric Isocyanic Acid

**DOI:** 10.1289/ehp.119-a338a

**Published:** 2011-08-01

**Authors:** Carol Potera

**Affiliations:** Carol Potera, based in Montana, has written for *EHP* since 1996. She also writes for *Microbe*, *Genetic Engineering News*, and the *American Journal of Nursing*.

Isocyanic acid (chemically HNCO), a contaminant linked to cataracts and heart disease, has for the first time been measured in the atmosphere.^1^ The discovery was made by researchers at the National Oceanic and Atmospheric Administration (NOAA) who designed a new detection system to measure HNCO in environments where conventional methods could not previously detect it.

Study leader James Roberts and colleagues developed a specialized negative-ion proton-transfer chemical ionization mass spectrometer to measure organic acids emitted by wildfires^2^ [look for more information on health effects of wildfires in the September 2011 issue of *EHP*]. They tested the instrument on brush and tree branches burned in a test chamber, which generated levels of HNCO reaching 600 ppbv. Next they used the device on air samples collected in Boulder, Colorado, during the 2010 Fourmile Canyon wildfire and ambient air samples collected in downtown Los Angeles, California. These samples yielded HNCO concentrations up to 200 pptv and 100 pptv, respectively. No wildfires were burning near Los Angeles at the time, so “we assume isocyanic acid came from vehicle exhaust or photochemical reactions known to make it,” Roberts says.

The investigators also observed HNCO in laboratory samples of cigarette smoke but noted “the levels were too high for us to quantify with the . . . instrument configured in the ambient measurement mode.” Drawing on a surrogate pyrolysis study that found nearly all the urea in tobacco decomposes to HNCO during burning,^3^ the researchers calculated mainstream cigarette smoke may contain 40–140 ppmv HNCO.^1^ Urea is added to cigarettes to enhance flavor.

Urea is also used in selective catalytic reduction systems to break down toxic nitrogen oxides (NO_X_) in diesel exhaust into nitrogen and water. Roberts wants to measure HNCO emitted by these systems, which the European Union mandates for heavy-duty diesel trucks; a similar law is pending in California.^4^ “In trying to solve the problem of NO_X_ we could be increasing HNCO,” Roberts says.

The health effects of chronic exposure to environmental HNCO are unknown, although Roberts and colleagues note the concentrations they measured in smoke “cause carbamylation at physiologically significant levels.”^1^ In carbamylation, cyanate binds the amino acid lysine in proteins to form homocitrulline. Stanley Hazen, section head of Preventive Cardiology and Rehabilitation at the Cleveland Clinic, found that high blood levels of homocitrulline are a strong predictor of heart disease, especially in smokers, offering a possible mechanism linking smoking to atherosclerosis.^5^

“We assumed the exogenous source of cyanate in our study was smoking,” Hazen says. Now Roberts’ study suggests HNCO from air pollution also may increase cardiovascular disease risk. “Any degree of carbamylation has potential to be harmful,” Hazen says, although the levels required to raise cardiac and other health risks remain to be determined.
